# Enhancing cytotoxic and apoptotic effect in OVCAR-3 and MDAH-2774 cells with all-trans retinoic acid and zoledronic acid: a paradigm of synergistic molecular targeting treatment for ovarian cancer

**DOI:** 10.1186/1756-9966-29-102

**Published:** 2010-07-30

**Authors:** Bulent Karabulut, Burcak Karaca, Umut Varol, Ugur Muslu, Burcu Cakar, Harika Atmaca, Aslı Kısım, Selim Uzunoglu, Ruchan Uslu

**Affiliations:** 1 Division of Medical Oncology, Tulay Aktas Oncology Hospital, School of Medicine, Ege University, 35100, Bornova, Izmir, Turkey; 2 Section of Molecular Biology, Department of Biology, Faculty of Science and Arts, Celal Bayar University, 45140, Muradiye, Manisa, Turkey

## Abstract

**Background:**

Ovarian cancer is the most fatal gynecologic malignancies in the world. Although, platinum based treatments are widely used, the disease becomes treatment refractory within two years, and novel treatment options should be searched. All- trans retinoic acid (ATRA) induces growth arrest, differentiation and cell death in some types of cancer cells and its combination with various anticancer agents results in enhanced cytotoxicity. Zoledronic acid is a common bisphosphonate known for its anticancer effects beyond its current use in the treatment of cancer-induced bone disease. We aimed to investigate the possible additive/synergistic effect of both agents in OVCAR-3 and MDAH-2774 ovarian cancer cell lines, since both agents show superiority to conventional cytotoxics in terms of adverse events.

**Methods:**

XTT cell proliferation assay was used for showing cytotoxicity. For verifying apoptosis, both DNA Fragmentation by ELISA assay and caspase 3/7 activity measurement were used. OligoGeArray^® ^which consists of 112 apoptosis related genes was used to elucidate the genetic changes within cancer cells. To validate our oligoarray results, quantitative real-time PCR was performed on four selected genes that were maximally effected by the combination treatment: lymphotoxin beta receptor (LTBR), myeloid cell leukemia-1 (MCL-1), tumor necrosis factor receptor superfamily, member 1A (TNFRSF1A), TNFRSF1A-associated death domain protein (TRADD).

**Results:**

We demonstrated that a novel combination of ATRA and zoledronic acid is a strong inducer of apoptotic related cell death in both ovarian cancer cells. While the combination therapy significantly induced proapoptotic genes such as tumor necrosis factor receptor superfamily (TNFRSF), TRADD and caspase 4, some of the antiapoptotic genes such as members of MCL-1, LTBR, BAG3 and Bcl-2 family members were inhibited.

**Conclusions:**

These are the preliminary molecular results of a novel combination treatment of ATRA and zoledronic acid, with fewer side effects as compared to conventional cytotoxic agents. With additional experimental analysis, it may serve as a good option for the treatment of refractory and elderly ovarian cancer patients, for whom there exists very limited choice of treatment.

## Background

Ovarian cancer is the most fatal gynecologic malignancy in the world [1]. Standard chemotherapy for previously untreated patients with advanced ovarian cancer consists of the combination of a taxane and a platinum compound. However, the disease becomes chemo-refractory within approximately two-years, and second-line treatment options do not provide significant survival advantage [2]. Thus, novel treatment approaches are needed to be investigated for this era.

Retinoids include both natural and synthetic derivatives of vitamin A. In the cell, they act by binding nuclear receptors that function as retinoid-dependent transcriptional factors, including the RAR and RXR receptors [3,4]. All- trans retinoic acid (ATRA), a natural derivative of vitamin A, induces growth arrest, differentiation and cell death of different types of cancer cell lines in vitro [5,6]. In the literature, there is a body of evidence that ATRA enhances the cytotoxic effects of chemotherapeutic agents [7-10]. There are some encouraging data from preclinical trials that have demonstrated the efficacy of using retinoids and cytotoxics in combination [11-13]. 

Zoledronic acid, a third-generation bisphosphonate, inhibits osteoclastic resorptive activity partly through inhibition of farnesyl-diphosphate synthase and protein prenylation [14]. Though it is mainly used for the treatment of cancer-induced bone disease, the promising findings coming from substantial amount of preclinical and early clinical evidence on the cytotoxic effect of zoledronic acid have led to several ongoing studies that will ascertain the benefit of zoledronic acid, itself, may act as a new antitumor agent in some human cancers [14,15]. Latest trials have demonstrated that zoledronic acid also has diverse anti-tumor effects via multiple mechanisms [16,17]. In preclinical models, bisphosphonates directly inhibit tumour growth and angiogenesis. Two recent clinical trials, ABCSG13 and Z/Zo-FAST have shown a disease-free survival benefit with zoledronic acid in women receiving adjuvant endocrine therapy [18,19]. Thus, it has been discussed to be used in the extended adjuvant treatment of early breast cancer as a new, promising anti cancer drug.

The wide spectrum toxic side effects of cytotoxic treatment as well as drug resistance occur to be important limitations of management of ovarian cancer, thus new treatment approaches are needed. Based on the knowledge of ATRA may work as enhancer of cytotoxic effect when added to other drugs, we investigated the possible additive/synergistic combination of ATRA with zoledronic acid in human ovarian cancer cell lines, OVCAR-3 and MDAH-2774. Since both of the agents have much more tolerable side effects as compared to conventional cytotoxic drugs, we searched for if this new combination might be a hope for elderly ovarian cancer patients.

Ovarian cancer cell lines can potentially overcome the experimental limitations inherent in both the animal models of ovarian cancer and the primary cloning of human ovarian cancer specimens. The availability of adequate quantities of proliferating cancer cells would also facilitate the identification of potentially clinically useful dose-response relationships with anti-cancer drugs as well as a model system in which to compare the cytotoxicity of drug analogues. OVCAR-3 is a highly metastatic, drug resistant human ovarian carcinoma cell line, and thus it is an ideal model to study the effects and mechanisms of various anticancer agents [20]. Besides, MDAH-2774 represents an example of slow-growing tumor type and was chosen a reciprocal experimental effect when used with OVCAR-3.

## Methods

### Cell lines and reagents

Human ovarian OVCAR-3 and MDAH-2774 cancer cells were obtained from ICLC (Genova, Italy). The cells were grown as monolayers in adherent cell lines and were routinely cultured in RPMI 1640 supplemented with 10% heat-inactivated fetal bovine serum (FBS), 1% L-glutamine, 1% penicillin-streptomycin in 75 cm^2 ^polystyrene flasks (Corning Life Sciences, UK) and maintained at 37°C in a humidified atmosphere with 5% CO_2_. Cell culture supplies were obtained from Biological Industries (Kibbutz Beit Haemek, Israel). ATRA was obtained from Sigma Chemical Co (USA). Zoledronic acid was a generous gift from Novartis Pharmaceuticals Inc. (Basel, Switzerland). The stock solution of ATRA was prepared in DMSO (43 mM) and, zoledronic acid (10 mM) was prepared in distilled water. The DMSO concentration in the assay did not exceed 0.1% and was not cytotoxic to the tumor cells. All other chemicals, unless mentioned, were purchased from Sigma.

### XTT cell viability assay

After verifying cell viability using trypan blue dye exclusion test by Cellometer automatic cell counter (Nexcelom Inc., USA.), cells were seeded at approximately 1×10^4^/well in a final volume of 200 μl in 96-well flat-bottom microtiter plates with or without various concentrations of drugs. Plates were incubated at 37°C in a 5% CO_2 _incubator for the indicated time periods. At the end of incubation, 100 μl of XTT (2,3-bis (2-methoxy-4-nitro-5-sulfophenyl)-5-[(phenylamino) carbonyl]-2H-tetrazolium hydroxide) (Roche Applied Science, Mannheim, Germany) was added to each well, and plates were incubated at 37°C for another 4 hours. Absorbance was measured at 450 nm against a reference wavelength at 650 nm using a microplate reader (Beckman Coulter, DTX 880 Multimode Reader). The mean of triplicate experiments for each dose was used to calculate the IC_50 _and the combination index (CI) values.

### Evaluation of apoptosis

Apoptosis was evaluated by enzyme-linked immunosorbent assay (ELISA) using Cell Death Detection ELISA Plus Kit (Roche Applied Science, Mannheim, Germany) and verified by measuring caspase 3/7 enzyme activity (Caspase-Glo 3/7 Assay, Promega, Madison, WI). Assays were described in our previous study [21].

### Examination of the expression levels of apoptotic genes by oligoarray method

Expression levels of apoptosis specific genes were examined by Human Apoptosis OligoGEArray^® ^(SuperArray, Frederick, MD). The OligoGEArray Human Apoptosis microarray profiles the expression of 112 genes involved in apoptosis. This array includes tumor necrosis factor (TNF) ligands and their receptors, members of the bcl-2 gene family, caspases and some other important apoptosis-related genes. Briefly, total RNA was extracted from cell samples using an Array Grade Total RNA isolation kit (SuperArray, Frederick, MD) and quantitated by UV spectroscopy using a biophotometer. The integrity and quality of isolated RNA was determined by running the RNAs on agarose gel electrophoresis. cDNA was labeled from total RNA with Biotin 16-dUTP and the GEArray^® ^TM Amp Labeling-LPR Kit (SuperArray, Frederick, MD) according to manufacturer's instructions. The biotin-labeled cDNA was than added to the membrane and hybridized overnight to Human Apoptosis OligoGEArray^® ^as stated by the manufacturer. Signal detection was achieved by exposure to CDP-Star alkaline phosphatase chemiluminescent substrate (SuperArray, Frederick, MD). An image was processed using Kodak^® ^Gel Logic 1500 Imaging System and analyzed with the GEArray Analyzer Software. Experiments were repeated thrice using RNA extracted from three different cultures.

### Real time quantitative RT-PCR (qRT-PCR) assay

To validate our oligoarray results, quantitative real-time PCR was performed on four selected genes that were maximally effected by the combination treatment: lymphotoxin beta receptor (LTBR), myeloid cell leukemia-1 (MCL-1), tumor necrosis factor receptor superfamily, member 1A (TNFRSF1A), TNFRSF1A-associated death domain protein (TRADD). Glyceraldehyde-3-phosphate dehydrogenase (GAPDH) was used as a positive control by using Real-Time™ qPCR Primer Assay (SABioscience, Frederick, MD) on Light Cycler 480 instrument (Roche Applied Science, Mannheim, Germany). Total RNA of 4 μg was extracted from cell samples using an Array Grade Total RNA isolation kit (SuperArray, Frederick, MD) and quantitated by UV spectroscopy using a biophotometer. The integrity and quality of isolated RNA was determined by running the RNAs on agarose gel electrophoresis. PCR reaction mix was prepared 25 μl final volume containing 12,5 μl RT2 SYBR Green qPCR Master Mix, 10,5 μl DNAase-RNaseFree water, 1,0 μl gene-specific 10 μM PCR primer pair stock and finally 1,0 μl diluted cDNA samples for each primer (SABioscience). Universal cycling conditions (10 min at 95°C, 15 s at 95°C, 1 min 60°C for 40 cycles) were carried out. The melting protocol consisted of 95°C for 1 minutes and a continuous fluorescense reading from 65°C to 95°C at 30 acquisitions per degree and 1°C rising per second. Data normalization and analysis an endogenous control, GAPDH present on the PCR was used for normalization. Each replicate cycle threshold (CT) was normalized to the average CT of endogenous control on a sample basis. The comparative CT method was used to calculate the relative quantification of gene expression. The following formula was used to calculate the relative amount of the transcripts in the drugs treated samples and the control group, both of which were normalized to the endogenous control. ΔΔCT = ΔCT (drugs treated) - ΔCT (control) for RNA samples. ΔCT is the log^2 ^difference in CT between the target gene and endogenous controls by subtracting the average CT of controls from each replicate. The fold change for each treated sample relative to the control sample = 2^-ΔΔCT^.

### Statistical analysis

All experiments were conducted in triplicate and the results expressed as the mean ± (sd), with differences assessed statistically p values determined by Student's *t*- test. p < 0.05 was accepted as significant. Median dose effect analysis, a measure of synergism or antagonism, was determined by the method of Chou and Talalay, using their computer program (Biosoft CalcuSyn, Ferguson, MO, USA) to assess drug interaction. We chose this method because it takes into account both the potency of each drug or combination of drugs and the shape of dose-effect curve. CalcuSyn software which is based on this method was used to calculate the CI. Synergy, additivity and antagonism were defined as CI < 1, CI = 1, CI > 1, respectively, where CI ≤ 0.5 characterizes strong synergy. For this analysis, concentrations of ATRA and zoledronic acid were chosen as clinically achievable concentrations and below the IC_50 _values [22].

## Results

### Effect of either single ATRA or zoledronic acid on the viability of OVCAR-3 and MDAH-2774 cells

**Figure 1:**
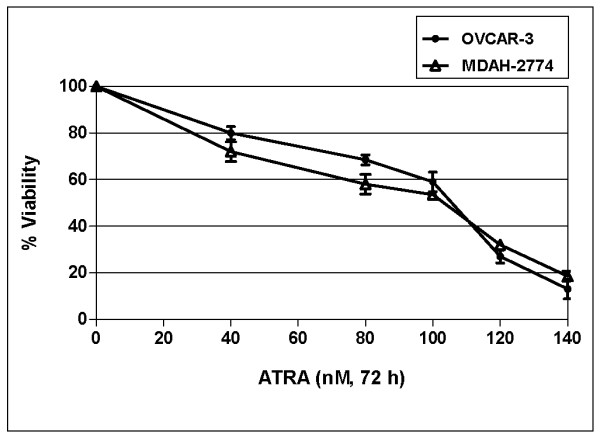
**Effect of ATRA on viability of OVCAR-3 and MDAH-2774 cells at 72 h in culture**. The data represent the mean of three different experiments (p < 0.05).

To evaluate the effects of ATRA on the viability of human ovarian cancer cells, OVCAR-3 and MDAH-2774 cells were exposed to increasing concentrations of ATRA (40 to 140 nM) for 24, 48 and 72 h, and XTT cell viability assay was performed. ATRA decreased cell viability in a time- and dose dependent manner both in OVCAR-3 and MDAH-2774 cells (data not shown). As shown in figure 1, there were 20-, 41-, and 73% decrease in cell viability of OVCAR-3 cells exposed to 40-, 100-, and 120 nM of ATRA, respectively, when compared to untreated controls at 72 h (p < 0.05). In addition, there were there were 28-, 49.5-, and 58% decrease in cell viability of MDAH-2774 cells exposed to 40-, 100-, and 120 nM of ATRA, respectively, when compared to untreated controls at 72 h (figure 1) (p < 0.05). Highest cytotoxicity was observed at 72 h and IC_50 _values of ATRA were calculated from cell proliferation plots and found to be 85 and 82 nM in OVCAR-3 and MDAH-2774 cells, respectively.

**Figure 2:**
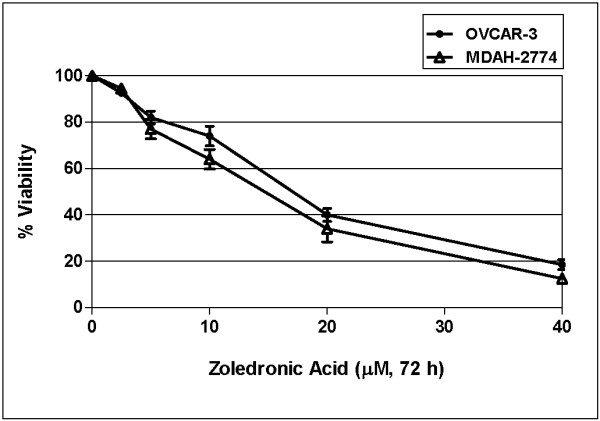
**Effect of zoledronic acid (ZA) on viability of OVCAR-3 and MDAH-2774 cells at 72 h in culture**. The data represent the mean of three different experiments (p < 0.05).

We also examined the effect of zoledronic acid on OVCAR-3 and MDAH-2774 cells. Cells were exposed to increasing concentrations of zoledronic acid (2.5- to 40 μM) for 24, 48 and 72 h. There were 18-, 26-, and 60% decreases in cell viability of OVCAR-3 cells exposed to 5-, 10-, and 20 μM of zoledronic acid, respectively, when compared to untreated controls at 72 h (figure 2) (p < 0.05). In MDAH-2774 cells, there were 22-, 35-, and 65% decreases in cell viability of MDAH-2774 cells exposed to 5-, 10-, and 20 μM of zoledronic acid, respectively (figure 2) (p < 0.05). Highest cytotoxicity was observed at 72 h and IC_50 _values of zoledronic acid in OVCAR-3 and MDAH-2774 cells were calculated from cell proliferation plots and were found to be 15.5 and 13 μM, respectively.

### ATRA and zoledronic acid combination treatment in OVCAR-3 and MDAH-2774 cells

**Figure 3:**
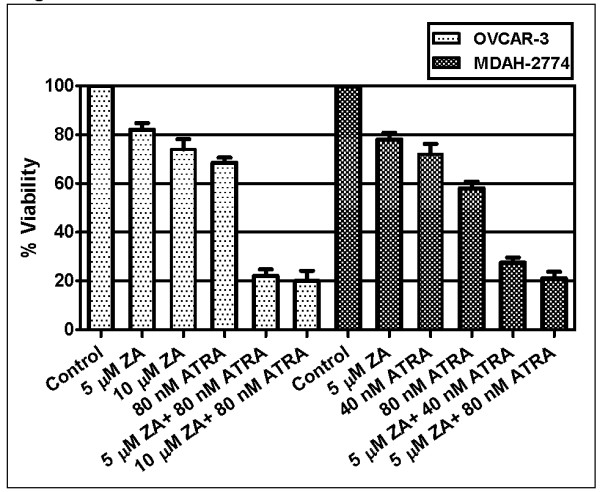
**Synergistic cytotoxic effects of ATRA and zoledronic acid (ZA) combination on viability of OVCAR-3 and MDAH-2774 cells at 72 h in culture *(p < 0.05)***.

To study the possible synergistic/additive effects of ATRA and zoledronic acid combination, OVCAR-3 and MDAH-2774 cells were exposed to different concentrations of each agent alone, and in combination of both for 24, 48 and 72 hours. The synergism or additivity was calculated via CI by using Biosoft Calcusyn Program. Combination of different concentrations of ATRA and zoledronic acid were evaluated at different time points (data not shown). Results showed synergistic toxicity in both ovarian cancer cells, OVCAR-3 and MDAH-2774, at 72 h, as compared to any agent alone as shown in table 1. Our results indicate that 80 nM ATRA and 5 μM zoledronic acid show 32%- and 18% decrease, respectively, in cell viability of OVCAR-3 cells but the combination of both resulted in 78% decrease in cell viability (figure 3). In MDAH-2774 cells, 40 nM ATRA and 5 μM zoledronic acid show 28%- and 22% decrease, respectively, in cell viability of MDAH-2774 cells but the combination of both resulted in 74% decrease in cell viability (figure 3).

**Table 1:** Combination index values

OVCAR-3		
**Concentration of Drugs**	**CI value**	**Interpretation**
Zoledronic acid (5 μM) + ATRA (80 nM)	0.688	Synergism
Zoledronic acid (10 μM) + ATRA (80 nM)	0.705	Synergism
**MDAH-2774**		
**Concentration of Drugs**	**CI value**	**Interpretation**
Zoledronic acid (5 μM) + ATRA (40 nM)	0.010	Synergism
Zoledronic acid (5 μM) + ATRA (80 nM)	0.009	Synergism

Combination index values of ATRA and zoledronic acid alone and in combination in OVCAR-3 and MDAH-2774 cells. CI values were calculated from the XTT cell viability assays. The data represent the mean of three independent experiments

CI ^a^: Combination index

ATRA*: All trans retinoic acid

The concentrations for each agent found to be synergistic in OVCAR-3 and MDAH-2774 cells are presented in table 1.

### Effects of the sequential treatment

The previous findings demonstrated that tumor cells with ATRA and zoledronic acid resulted in significant synergism at 72 h. Sequential administration of the drugs were carried out to see if either of these drugs enhance the other one's effect and to understand whether the synergism depended on which agent applied first. We examined the effect of sequential treatment of OVCAR-3 and MDAH-2774 cells with either ATRA or zoledronic acid and subsequent treatment with the second agent. Pretreatment of tumor cells with ATRA for 36 h and wash and then treatment for an additional 36 h with zoledronic acid resulted in synergistic cytotoxicity in OVCAR-3 and MDAH-2774 cells. Also, pretreatment of tumor cells with zoledronic acid for 36 h and wash and then treatment for an additional 36 h with ATRA resulted in synergistic cytotoxicity in OVCAR-3 and MDAH-2774 cells (data not shown). So, synergistic cytotoxicity was observed no matter which agent applied first in both cells.

### Combination treatment induced apoptosis in a synergistic manner

#### a) DNA Fragmentation

To examine the induction of apoptosis in response to ATRA or zoledronic acid and combination of both in ovarian cancer cells, we incubated these cells in the presence of the agents alone or in combination of both for 72 hours and then we quantified the levels of mono-oligo nucleosome fragments by Cell Death Detection Kit (Roche Applied Science, Mannheim, Germany). Our results clearly showed that both ATRA and zoledronic acid alone induced apoptosis in a dose-dependent manner but the exposure to combination of both agents resulted in synergistic induction of apoptosis by DNA fragmentation analysis.

**Figure 4:**
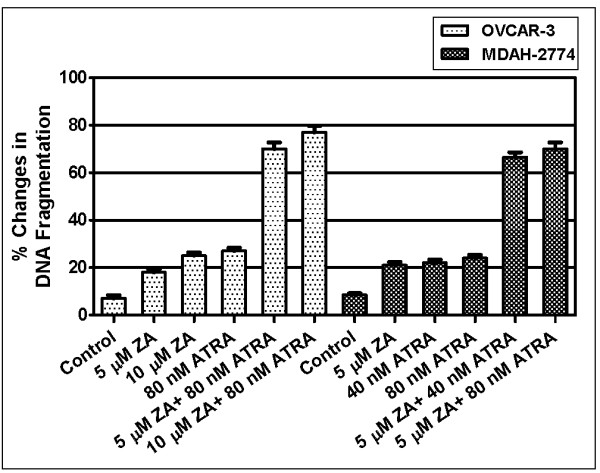
**Apoptotic effects of ATRA and zoledronic acid (ZA) alone or in combination in OVCAR-3 and MDAH-2774 cells through DNA fragmentation analyses *(p < 0.05)***.

As shown in figure 4, there were 2.7- or 1.8- fold increases in DNA fragmentation in 80 nM ATRA or 5 μM zoledronic acid exposed OVCAR-3 cells, respectively, as compared to untreated controls, while the combination of both resulted in 7 fold increase in DNA fragmentation (p < 0.05). In MDAH-2774 cells, there were 2.0- or 1.9- fold increase in DNA fragmentation in 40 nM ATRA or 5 μM zoledronic acid exposed MDAH-2774 cells respectively, as compared to untreated controls, while the combination of both resulted in 6.2 fold increase in DNA fragmentation (figure 4) (p < 0.05). These doses were chosen to put in the figure, since they represent the most demonstrative synergistic dose-dependent effect of the combination.

#### b) Caspase 3/7 enzyme activity

**Figure 5:**
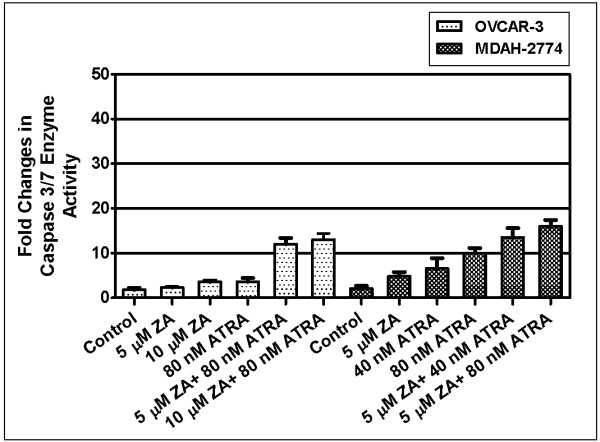
**Percentage changes in caspase 3/7 enzyme activity in ATRA and zoledronic acid combination or any agent alone exposed OVCAR-3 and MDAH-2774 cells *(p < 0.05)***.

Caspases are commonly referred to as hangmans of apoptosis. The activation of caspases is an evidence of apoptosis in cells. In order to confirm the apoptotic effects of combination treatment in OVCAR-3 cells, we examined the changes in caspase 3/7 enzyme activity. The results revealed that there was a dose dependent increase in caspase 3/7 enzyme activity in ATRA or zoledronic acid in OVCAR-3 cells (data not shown). Specifically, OVCAR-3 cells exposed to 80 nM ATRA or 5 μM zoledronic acid showed 2.8- or 1.7- fold increases in caspase 3/7 enzyme activity, respectively, as compared to untreated controls, while their combination resulted in 6.6- fold increases in caspase-3/7 enzyme activity (figure 5) (p < 0.05). MDAH-2774 cells exposed to 40 nM ATRA or 5 μM zoledronic acid showed 3.1- or 2.2- fold increases in caspase 3/7 enzyme activity, respectively, as compared to untreated controls, while their combination resulted in 6.1- fold increases in caspase-3/7 enzyme activity (figure 5) (p < 0.05).

#### Oligoarray and RT-PCR analyses of apoptosis-related genes in OVCAR-3 cells by the combination treatment

We used apoptosis specific oligoarray to examine the changes in expression levels of mRNAs of the apoptosis related genes in response to ATRA and zoledronic acid treatment in OVCAR-3 cells as compared to untreated controls. Based on the IC_50 _results of each agent in OVCAR-3 and MDAH-2774 cells, OVCAR-3 cancer cells were found to be more chemorefractory. Thus, we have chosen OVCAR-3 cell line to study the mechanistic rationale of apoptosis with this combination. For this experiment, we have applied the doses of 80 nM ATRA and 5 μM zoledronic acid for oligoarray experiments. These doses were chosen because they are much more less than the IC_50 _doses of each agent and weak inducers of apoptosis in OVCAR-3 cells, and thus letting the oligoarray results not to be shaded by strong apoptotic effect. Three repeated experiments were carried out and the results showed that there were 6.8-, 4.9- and 4.8- fold increase in TNFRSF 1A, 10B and TNFRSF 1A-associated death domain (TRADD) mRNA levels in OVCAR-3 cells when treated with combination of ATRA and zoledronic acid, as compared to any agent alone (table 2) (p < 0.05). Moreover, proapoptotic members of Bcl-2 family (i.e BNIP3) were also shown to be induced whereas the antiapoptotic members of the same family (i.e BCL2L1, BCL2L12, BCL2L13) were inhibited by the treatment.

**Table 2:** Fold changes in apoptosis related genes by OligoArray in OVCAR-3 cells

	Fold Change in OVCAR-3 cells		
**Gene Symbol**	**ATRA** **(80 nM)**	**Zoledronic Acid** **(5 μM)**	**Combination**

**BCL2L-1 (BCL-xL)**	-1.8	-2.1	-4.0
**BCL2L12**	-1.3	-1.5	-3.1
**BCL2L13**	-1.3	-2.6	-7.0
**BNIP3**	+1.9	+2.4	+3.9
**TNFRSF1A**	+1.5	+3.6	+6.8
**TNFRSF10B**	+1.6	+3.4	+4.9
**TRADD**	+1.3	+1.2	+4.8
**CASP4**	+1.2	+1.4	+3.2
**MCL-1**	-2.2	-1.6	-3.3
**BAG3**	-1.0	-1.0	-3.1
**LTBR**	-1.4	+2.5	-4.9

*p < 0.05

In contrary, mRNA levels of lymphotoxin beta receptor (LTBR), myeloid cell leukemia-1 (MCL-1) and BCL2-associated athanogene 3 (BAG3) were reduced by the combination treatment by 4.9-, 3.3- and 3.1- fold decrease, respectively, as compared to each of the single agent (table 2) (p < 0.05). The genes mentioned above are responsible for resistance to apoptosis in many types of human cancer cells, thus the reduction of mRNA levels of these genes point out that the synergistic combination treatment is effective on inducing apoptosis in OVCAR-3 cells.

The results of the oligoarray data were validated by quantitative real-time RT-PCR, using three genes relevant to apoptosis: I) Proapoptotic genes: TNFRS1A and TRADD which are both the key genes that start the apoptotic cascade when induced in cancer cells, II) Antiapoptotic genes: MCL-1 and LTBR which both have versatile roles in regulation of apoptosis and cell cycle progression.

For all these genes, the levels of expression observed by quantitative RT-PCR highly correlated with the data obtained by oligoarray analysis. TNFRS1A and TRADD are found to be upregulated by 8.3- and 3.5- fold by combination treatment, whereas MCL-1 and LTBR were downregulated by 4.9- and 3.6- fold, respectively, as compared to any agent alone (table 3) (p < 0.05).

**Table 3:** OligoArray and RT-PCR comparison of fold changes in apoptosis related genes

OVCAR-3 Cells (80 nM ATRA+5 μM Zoledronic Acid)		
	**OligoArray Analysis**	**RT-PCR Analysis**

**TNFRSF1A**	+6.8	+8.3
**TRADD**	+4.8	+3.5
**MCL-1**	-3.3	-4.9
**LTBR**	-4.9	-3.6

Comparing fold changes in apoptosis related genes in OVCAR-3 cells by OligoArray and RT-PCR analyses after each drug exposure. Both results showed high correlation with each other (p < 0.05).

## Discussion

Despite to response to some effective therapeutic approaches, decreased ability to undergo apoptosis by malignant evolution of cancer cells is one of the main problems of daily oncology practice [23]. Selective strategies to manipulate cancer cells towards apoptosis rather than normal cells in the tissue are emerging as new potential therapeutics. Thus, apoptosis inducer agents that are non-toxic for healthy cells are promising agents of future cancer treatment.

Our preliminary preclinical data demonstrated that the combination of ATRA and zoledronic acid has synergistic cytotoxic effect in OVCAR-3 and MDAH-2774 ovarian cancer cells as compared to any agent alone. Since ATRA is known to potentiate the cytotoxicity of some conventional chemotherapeutics, this enhancement effect has also observed in combination with zoledronic acid for ovarian cancer cells in our experiments. In addition, it was also shown that this combination induces apoptosis synergistically in ovarian cancer cells through activation of caspases and induction of DNA fragmentation. We have also shown that the combination of ATRA and zoledronic acid significantly alters the levels of some important apoptosis-related molecules in OVCAR-3 cells, both by oligoarray and RT-PCR analyses.

By oligoarray analysis, we have shown that the mRNA levels of TNFRSF genes are induced by the exposure of the both agents. In the cancer cell, the death-signaling pathway begins from the interaction of TNFRs with their specific ligands and this pathway subsequently initiates apoptosis via activation of caspases and downstream of protein cascade leading to cell death [24,25]. Among these receptors, TNFRSF 1A is one of the most popular receptor since its ligand, tumor necrosis factor-α (TNF-α), takes roles in wide range biological activities associated with apoptosis/cell survival in many type of cells [26]. After TNF-α recruits to its receptor, an interaction between cytoplasmic death domain of TNFR 1A and the adaptor molecule TNFRSF 1A-associated death domain protein (TRADD) takes place. We have shown that mRNA levels of TNFRS 1A, 10B and TRADD were increased by the combination treatment with ATRA and zoledronic acid, suggesting the cell was under strong apoptotic stimuli. Besides, caspase 4 mRNA levels were also up-regulated by the same combination. In the literature, there is a body of evidence showing that exposure to ATRA results in the upregulation of cell surface expression of TNFRs in some type of cancer cells [27]. Thus, exposure of cancer cells with ATRA and zoledronic acid combination results in strong apoptotic stimuli through TNFRs.

The Bcl-2 family proteins are central regulators of apoptosis because they integrate diverse survival and death signals that are generated outside and inside the cell [28,29]. The combination treatment in our study resulted downregulation of some important Bcl-2 antiapoptotic members (Bcl-2 L1, Bcl-2 L12, Bcl-2 L13) whereas an induction in proapoptotic family member (the Bcl-2/adenovirus E1B-19K interacting protein BNIP3) was observed. Besides, there was a downregulation of mRNA levels of BAG3 with ATRA and zoledronic acid combination. BAG-3 (Bis) has also been reported to associate with the anti-apoptotic protein Bcl-2 [30]. Functional analysis revealed that BAG-3 itself exerts only weak anti-apoptotic activity, but acts synergistically with Bcl-2 in preventing Bax-induced and FasL/Fas-mediated apoptosis

mRNA levels of MCL-1 and LTBR genes were also reduced by the combination treatment. The MCL-1 gene was discovered incidentally as an induction gene in myeloblastic leukemia cell differentiation about a decade ago and proved to be a member of the emerging Bcl-2 gene family [31]. LTBR is also a very good example of two-way functioning molecules. LTBR is a member of TNFRSF that regulate cell survival or death through activation of nuclear factor kappa B (NF-kappaB). In some studies, it was clearly shown that by binding LTBR with some specific or oligo-sense antibodies resulted in decreased tumor growth and increased apoptosis in tumor cells [32-34].

Our oligo array results were also verified with RT- PCR assay, and the results highly correlated with each other. Of these genes, TNFRSF1A and TRADD were found to be upregulated since they work as the trigger molecules of the apoptotic cascade in cancer cells whereas antiapoptotic genes MCL-1 and LTBR were found to be downregulated.

## Conclusions

Retinoids are widely investigated as the enhancers of cytotoxic agents in cancer treatment. Since they do not have any significant toxic side effect, they represent good candidates for combination treatment. Zoledronic acid, far beyond its effect on bone turn over, has presented some novel antitumoral activity even in the adjuvant treatment of cancer. So, in conclusion, these findings provide basic molecular information for further investigation on the mechanisms by which ATRA and zoledronic acid exert their pleiotropic effects in ovarian cancer cells. However, our study has some important limitations; first we do not yet provide in vivo results of our combination treatment, which will certainly be very helpful to support our data. Latter, our experiments have been tested only in ovarian cancer cells, and should further be validated in normal ovarian cells. Further in-depth investigations should be done to confirm the efficacy of this potentially new treatment for ovarian cancer.

## Abbreviations

ATRA: All- trans retinoic acid; ZA: zoledronic acid; RAR: Retinoic acid receptor; RXR: Retinoid X receptor; XTT: 2,3-bis (2-methoxy-4-nitro-5-sulfophenyl)-5-[(phenylamino) carbonyl]-2H-tetrazolium hydroxide; ELISA: enzyme-linked immunosorbent assay; ABTS: 2,29-Azino-di-[3-ethylbenzthiazolinesulfonate] diammonium salt; CI: Combination index; IC50: Concentration of inhibition 50% TRADD: TNFR1-associated death domain; TNFRSF: Tumor necrosis factor receptor super family; TNF-α: Tumor necrosis factor-α BNIP3: The Bcl-2/adenovirus E1B-19K interacting protein; BCL2L1: BCL2-like 1; BCL2L12: BCL2-like 12; BCL2L13: BCL2-like 13; LTBR: Lymphotoxin beta receptor; BAG3: BCL2-associated athanogene 3; MCL-1: Myeloid cell leukemia-1; Bax: BCL2-associated X protein; Bak: Bcl-2-like protein 7; NF-kappaB: Nuclear factor kappa B.

## Competing interests

The authors declare that they have no competing interests.

## Authors' contributions

BK carried out cytotoxicity experiments, and participated in the drafted manuscript, BK participated in the design of the study, UV performed statistical analysis, UM carried out molecular genetic studies, BC carried out cytotoxicity experiments, HA carried out apoptosis experiments, AK carried out apoptosis experiments, and molecular genetic studies, SU participated in design of the study, RU conceived of the study, and participated in its design and coordination. All authors read and approved the final manuscript.
